# SARS-CoV-2 challenge studies: ethics and risk minimisation

**DOI:** 10.1136/medethics-2020-106504

**Published:** 2020-09-25

**Authors:** Susan Bull, Euzebiusz Jamrozik, Ariella Binik, Michael J Parker

**Affiliations:** 1 The Ethox Centre & Wellcome Centre for Ethics and the Humanities, Nuffield Department of Population Health, University of Oxford, Oxford, Oxfordshire, UK; 2 Monash Bioethics Centre, Monash University, Melbourne, Victoria, Australia; 3 Department of Philosophy, McMaster University, Hamilton, Ontario, Canada; 4 The Ethox Centre, University of Oxford, Oxford, UK

**Keywords:** ethics, scientific research, research ethics, clinical trials

## Abstract

COVID-19 poses an exceptional threat to global public health and well-being. Recognition of the need to develop effective vaccines at unprecedented speed has led to calls to accelerate research pathways ethically, including by conducting challenge studies (also known as controlled human infection studies (CHIs)) with SARS-CoV-2 (the virus which causes COVID-19). Such research is controversial, with concerns being raised about the social, legal, ethical and clinical implications of infecting healthy volunteers with SARS-CoV-2 for research purposes. Systematic risk evaluations are critical to inform assessments of the ethics of any proposed SARS-CoV-2 CHIs. Such evaluations will necessarily take place within a rapidly changing and at times contested epidemiological landscape, in which differing criteria for the ethical acceptability of research risks have been proposed. This paper critically reviews two such criteria and evaluates whether the use of effective treatment should be a necessary condition for the ethical acceptability of SARS-CoV-2 CHIs, and whether the choice of study sites should be influenced by COVID-19 incidence levels. The paper concludes that ethical evaluations of proposed SARS-CoV-2 CHIs should be informed by rigorous, consultative and holistic approaches to systematic risk assessment.

## Introduction

The current pandemic of COVID-19, caused by SARS-CoV-2, poses an extraordinary threat to global health and well-being. In response, the current scale of COVID-19 research is unprecedented and there is international recognition of the urgent need to develop and distribute safe and effective vaccines globally.^1^ Vaccine development typically takes 10 to 20 years, including lengthy trials with human participants for the collection of sufficient evidence about safety and efficacy. The exceptional pace of COVID-19 vaccine research has resulted in the commencement of early human trials with vaccine candidates, accompanied by calls to consider the value of conducting challenge studies (also known as controlled human infection studies (CHIs)) with SARS-CoV-2 to further accelerate and inform vaccine development pathways.^2–4^

CHIs involve intentionally exposing participants to pathogens to study mechanisms of infection and disease and/or the efficacy of experimental vaccines or treatments. While such studies have previously received ethical approval and played an important role in vaccine development pathways for diseases, such as typhoid, cholera and malaria, the use of intentional exposure always requires careful consideration and rigorous ethical review.^5^ In the context of the COVID-19 pandemic, the ethical acceptability of conducting SARS-CoV-2 CHIs has been the subject of considerable interest and debate in the popular press, social media and academic literature.^2 6–8^ Two recent consensus documents addressing the ethics of SARS-CoV-2 CHIs have neither sanctioned nor prohibited such research in principle, but instead highlighted ethical issues requiring careful consideration during the design and review of such studies within the pandemic research landscape.^9 10^ In doing so they drew on broader norms of research ethics and focused attention on the need for substantial social and scientific values; appropriate risk–benefit profiles; careful site and participant selection; rigorous engagement and consent processes; appropriate compensation; and effective review, oversight and coordination.

In practice, much of the recent debate about the ethics of SARS-CoV-2 CHIs has centred on whether the risks associated with such studies can be considered acceptable. In this paper we critically explore approaches to risk minimisation and reasonableness in SARS-CoV-2 CHIs with a particular focus on two claims: (1) that effective treatment is always a necessary condition for the ethical acceptability of such studies and (2) that the choice of study sites should be responsive to COVID-19 incidence levels.

## Reasonable and minimised risks

International norms and consensus standards for the ethics of research with human participants highlight the importance of systematic evaluation of research risks, and the need for such risks to be proportionate, reasonable and minimised.^11^ During pandemics, it is critical to consider how such standards should guide research conducted within challenging contexts, where a complex and evolving landscape of professional and ethical codes of practice are informing clinical, public health, humanitarian and research responses.^12^

Within the context of the COVID-19 pandemic, for example, systematic evaluations of research risks associated with SARS-CoV-2 CHIs would need to take place within a rapidly changing, and at times contested, evidentiary landscape.^13^ In addition, COVID-19 vaccine research pathways have resulted in clinical trials of vaccine candidates beginning rapidly, with some routine stages of vaccine development, which could reduce uncertainties about research risks, being omitted.^14^ Systematic risk evaluations will consequently require consideration of both heightened uncertainties associated with accelerated research pathways and the novelty of the disease.

Within this complex evidentiary landscape, multiple commentators have sought to provide insights to inform evaluations of the ethical acceptability of risks associated with potential SARS-CoV-2 CHIs. In the text below, we briefly outline competing accounts of reasonable risks in research, and how upper levels of acceptable risk have been framed in the context of CHIs and pandemics. Against this background, we review proposed approaches to risk minimisation, and critically appraise claims that the risks of SARS-CoV-2 CHIs can only be acceptably minimised if an effective treatment is available or if CHIs are conducted in high-incidence settings.

### What risks are reasonable?

There is no consensus within research ethics guidance about whether research with healthy volunteers should be subject to upper levels of acceptable risk. Commentators endorse contrasting approaches. Some argue that there should be no upper limits, provided that studies involve competent, healthy adults and additional conditions, including informed consent and high social value, are met.^15^ A contrasting approach endorses limits on upper levels of acceptable risk, justified on grounds including the basis of fiduciary relationships between researchers and participants, equal moral concern for people’s basic interests, or the significance of preserving public trust in research.^16^ Suggested thresholds to inform the review of research protocols include a 1% risk of death, or risks comparable to those posed by other altruistic activities such as living organ donation.^10^ In the context of CHIs, suggested risk limits include a minimal risk threshold,^17^ a higher threshold that rules out risks of irreversible, incurable or possibly fatal infections,^18^ and approaches drawing on broader norms restricting research risks in healthy volunteers.^10 16^

In the context of COVID-19 pandemic research, the possibility of accepting higher levels of risk than might otherwise be considered reasonable has been raised,^2^ including comparisons with high-risk activities.^4^ However, higher risk or more burdensome research is particularly controversial in populations or communities already facing undesirably high degrees of risk^11 16^ and the importance of maintaining scientific and ethical research standards during public health crises has been persuasively emphasised. So too has the critical importance of engaging with local stakeholders and communities about proposed CHIs, including evaluations of the reasonableness and acceptability of risks.^8–10 12 16 19^ Given the novelty of COVID-19 and uncertainties about its long-term health implications, the acceptability of risks of SARS-CoV-2 CHIs should be subjected to particularly careful and continual assessment, in consultation with communities and stakeholders, in light of changing evidence, and taking into account risk minimisation strategies discussed below.

### Risk minimisation

In addition to determining whether the risks of proposed SARS-CoV-2 CHIs fall within thresholds for reasonable risk, systematic evaluations must also address whether risks will be managed and minimised consistent with sound scientific practice. At present there is consensus that participant inclusion/exclusion criteria are likely to play a key role in risk minimisation.^3 9 10^ COVID-19 is associated with strong age-related mortality trends, with current evidence suggesting that infection in young adults (under 30 years of age) is associated with a hospitalisation risk of 0.6%–1% and a risk of death of around 0.007%–0.03%—much lower than in older adults.^20 21^ Such evidence suggests that risks may be minimised by the use of recruitment criteria focusing on young healthy adults with the lowest risk of severe disease and mortality.^2 10 22^ In addition to age-related inclusion criteria, multiple commentators have highlighted the importance of ensuring that research risks are further minimised by ensuring that SARS-CoV-2 are only conducted in sites with the capacity, resources and expert research teams to closely monitor and provide excellent care to research participants, and provide appropriate follow-up, among other measures.^2 3 9 10^

#### Clarifying the roles of treatment in risk minimisation

Given the importance of providing care to participants who might develop symptoms, prospective CHI researchers must regularly review current research into treatments for COVID-19 so that proven treatments can be provided where possible and appropriate.^3 9^ There is debate, however, about the role of specific curative treatments in the evaluation of risk minimisation strategies. One argument is that such treatments could potentially play an important role in risk minimisation strategies but may not be a necessary requirement for the SARS-CoV-2 CHIs to be ethically acceptable.^9^ A contrasting claim is that in the absence of a ‘safe, effective, approved treatment’ for COVID-19, the risks associated with SARS-CoV-2 CHIs are unacceptable, because they cannot be appropriately minimised.^8^

In exploring the normative and empirical basis for the claim that an effective treatment is a necessary requirement for SARS-CoV-2 CHIs, it is valuable to explore both the concept and potential role(s) of treatment in challenge studies. First, treatment may be understood in terms of ‘rescuing’ a patient who would otherwise die from severe COVID-19. Mortality caused by severe COVID-19 (and many other fatal infectious diseases) results from an inflammatory cascade—from which antivirals alone, for example, cannot ‘rescue’ a patient. Other treatments including those targeting inflammation may have some effect, but this is often limited—as demonstrated by the recent trial of dexamethasone which prevented approximately one death for every eight persons treated.^23^ Dexamethasone is nevertheless an example of a ‘safe, effective, approved treatment’ which does not constitute a reliable form of ‘rescue’ for all patients.

Second, different types of treatment for infectious diseases can be aimed at one or more of: treatment of asymptomatically infected people to prevent symptoms/disease (prophylaxis), treatment of people who develop mild symptoms/disease to prevent progression to severe disease (‘disease-modifying’ treatment) and/or treatment of severe cases to reduce mortality (such interventions would be closest to ‘rescue’, but, like dexamethasone, these are almost never 100% effective). In malarial CHIs, for example, risk minimisation strategies must focus on the prevention of severe disease, rather than ‘rescue’ from severe outcomes, because by the time a person develops severe malaria a good outcome cannot be guaranteed, even with highly effective antimalarial drugs. In contrast, influenza CHIs are conducted although influenza antivirals have at most weak evidence for prophylaxis, very doubtful evidence of disease-modifying effects and no evidence of being able to rescue patients from severe influenza (eg, influenza myocarditis or heart inflammation).^24^

The availability, roles and anticipated outcomes of effective treatments are core considerations in systematic risk evaluations in CHIs. A review of ongoing clinical research into relevant treatments, and their implications for risk management should be an integral component of SARS-CoV-2 CHI risk reviews. Treatment could play a vital role in risk minimisation strategies in SARS-CoV-2 CHIs. However, the normative claim that a safe, effective and approved treatment must be an ethical precondition of any SARS-CoV-2 CHIs requires further clarification in terms of the specific aim and potential magnitude of risk minimisation that such a treatment is expected to achieve. In doing so it is important neither to underestimate nor to overestimate the potential impact of such treatments, as a treatment may not be necessary (eg, if it is not anticipated to materially contribute to risk minimisation strategies) nor will it be sufficient (eg, because treatment is never 100% effective and must be complemented by other risk minimisation strategies) to effectively address the risk of study participation. Consequently the need for a safe and effective treatment should not be considered in isolation, but instead reviewed within the context of a rigorous and comprehensive approach to risk minimisation.

#### Background risk

In addition to the approaches to risk minimisation outlined above, some commentators have claimed that COVID-19 CHIs should only be conducted with participants with an especially high baseline probability of exposure to SARS-CoV-2 during or soon after the proposed CHIs, solely to minimise the additional risks posed by controlled infection during research.^3^ The normative and empirical justifications for requiring that risk evaluations of SARS-CoV-2 CHIs prioritise a specific aspect of risk minimisation require careful consideration.

In practice, even though a very high background probability of infection (eg, a 60% probability of being infected in the local community during or soon after the study) would reduce the relative risk of participation by a large percentage (eg, around 60%), this might only constitute a small absolute risk difference. This is because, as discussed above, the probability of serious outcomes of infection is already extremely low for young healthy adults—a fraction of 1%—meaning that the absolute risk difference would be around 60% of a fraction of 1%.^25^

In addition to questions about the normative and empirical role that such small absolute risk reductions should play in decision-making, the practical and ethical implications of conducting SARS-CoV-2 CHIs in contexts with high background risks of infection require careful review. In practice, there are relatively few sites and research teams with the existing expertise, experience and capacity to conduct rigorous SARS-CoV-2 CHIs and effectively minimise the risks of research for participants and third parties. Within such contexts, a preparation period is needed to establish whether such a trial can meet relevant ethical, regulatory and clinical standards and to develop meaningful engagement strategies and participatory processes with local stakeholders and communities to determine whether such research is acceptable. Given the unpredictability of incidence levels in the face of changing public health transmission control policies,^20^ any requirement that SARS-CoV-2 CHIs can only be conducted within high-incidence settings may render them infeasible.

Additionally, the need to conduct research to address public health priorities must be evaluated in conjunction with the importance of ensuring that such research does not adversely impact pandemic response efforts.^12 26^ High-incidence settings provide an ideal environment for conducting field trials of COVID-19 vaccine candidates, which are infeasible in low incidence settings. Consequently in so far as COVID-19 vaccine research resources are limited in a particular location, it may be more appropriate to prioritise field trial testing in locations with (predicted) high incidence, rather than CHIs.

In settings with a high incidence of COVID-19, it is also important to recognise that health systems may have been catastrophically overburdened, requiring value-laden decisions to be made about the allocation of scarce resources to treat all healthcare conditions, protect front-line workers and continue existing research programmes.^27 28^ Rigorous risk minimisation during SARS-CoV-2 CHIs will require specialist facilities and equipment for infection control, in addition to the expertise of highly experienced researchers, who are often also clinicians specialising in infectious disease. Such requirements have the potential to directly impact the capacity of local health systems to address current public health needs during the peaks of epidemic waves.

Finally, although the probability of needing critical care for young healthy adult CHI participants will need to be very low for the risks of SARS-CoV-2 CHIs to be considered reasonable, participants’ access to scarce life-saving resources, if required, is a critical element of risk minimisation strategies. While researchers have a clear responsibility to provide such care if required, in practice the provision of such care may require negotiation and access to additional clinical resources and professional expertise. Some commentators have suggested that SARS-CoV-2 CHI participants should be guaranteed access to such resources, irrespective of their scarcity.^3^ An alternative proposition suggests priority access to scarce life-saving resources should be given to research participants only if they have similar prognoses to patients who may also need such resources.^28^ If SARS-CoV-2 CHIs were to be conducted in high-incidence settings where active rationing of scarce life-saving resources has been implemented, the justifications for guaranteed or prioritised access for participants, and any anticipated implications such access will have for public health responses, would require careful evaluation.

Taken together, these factors suggest that it is not straightforwardly the case that it is more ethical to conduct CHIs in high-incidence rather than low-incidence settings. Instead it can be argued that the practical and ethical considerations above warrant a systematic and comprehensive approach to risk management which does not unduly prioritise reducing the marginal risks of participation resulting from conducting such research in the context of high background risk. Such an approach recognises the importance of due consideration of the complex research, public health, regulatory, social and ethical implications of selecting potential sites for SARS-CoV-2 CHIs, including the impact of conducting such studies on public health and research responses to the pandemic and additional population health needs.

## Conclusions

The justifications for, and ethical acceptability of, conducting SARS-CoV-2 CHIs within accelerated vaccine pathways continue to be a focus of considerable public and academic interest and debate. Within this context, systematic assessments of the reasonableness and minimisation of research risks associated with any proposed SARS-CoV-2 CHIs will take place within a complex, rapidly changing and at times contested epidemiological and ethical landscape. Such assessments must be rigorous, holistic and informed by engagement with local stakeholders and proposed approaches which seek to isolate and prioritise the moral importance of specific risk management strategies require critical review. In particular, while the potential value of providing effective curative treatments to SARS-CoV-2 CHI participants (if needed) is uncontroversial, the implications of the absence of such treatment on the ethical acceptability on these studies merit additional scrutiny. We additionally make the case that selecting sites for potential SARS-CoV-2 CHIs requires careful review of a range of regulatory, ethical, public health, social and practical considerations, in which approaches to risk management should not unduly prioritise small reductions of the marginal risks of participation arising from conducting such research in the context of high background risk.

## Data Availability

There are no data in this work.
